# Radiographic Examination of Recruits

**Published:** 1918-08

**Authors:** 


**Radiographic Examination of Recruits.** "From a study of 977 cases we believe that the following conclusions are justified:

"1. That it is practical to make X-Ray examinations of the chests of soldiers at the rate of two cases per minute.

"2. That while six men were used in this series, the number could easily be reduced to four, and only one of them need be a Roentgenologist.

"3. That one man can easily develop one hundred plates every three hours.

"4. That an individual can interpret plates at the rate of two per minute, leaving the rejected or doubtful cases for further study.

"5. That in the 977 cases examined we found 3.6% of the cases with definite tuberculosis, sufficient to disqualify them for military service, and an additional 3.2% with tuberculosis which will require further interpretation and observation to determine whether their tuberculosis should disqualify them or not."

(Signed) Lewis Gregory Cole,

Major M. R. C. U. S. A.

